# Addressing the migrant gap: maternal healthcare perspectives on utilising prevention of mother to child transmission (PMTCT) services during the COVID-19 pandemic, South Africa

**DOI:** 10.1080/16549716.2022.2100602

**Published:** 2022-08-15

**Authors:** Melanie A. Bisnauth, Ashraf Coovadia, Mary Kawonga, Jo Vearey

**Affiliations:** a School of Public Health Faculty of Health Sciences, University of the Witwatersrand, Johannesburg, South Africa; b School of Clinical Medicine, Faculty of Health Sciences, University of the Witwatersrand, Johannesburg, South Africa; c Charlotte Maxeke Johannesburg Hospital and Department of Community Health, School of Public Health, University of Witwatersrand, Johannesburg, South Africa; d African Centre for Migration and Society, Faculty of Social Sciences, University of Witwatersrand, Johannesburg, South Africa

**Keywords:** HIV/AIDS, COVID-19, PMTCT, migration, South Africa

## Abstract

**Background:**

The COVID-19 pandemic has interrupted the prevention of mother-to-child transmission of HIV (PMTCT) programming in South Africa. In 2020, it was estimated that there were 4 million cross-border migrants in South Africa, some of whom are women living with HIV (WLWH), who are highly mobile and located within peripheral and urban areas of Johannesburg. Little is known about the mobility typologies of these women associated with different movement patterns, the impact of the COVID-19 pandemic on mobility typologies of women utilising PMTCT services and on how changes to services might have affected adherence.

**Objective:**

To qualitatively explore experiences of different mobility typologies of migrant women utilising PMTCT services in a high mobility context of Johannesburg and how belonging to a specific typology might have affected the health care received and their overall experiences during the COVID-19 pandemic.

**Methods:**

Qualitative semi-structured interviews with 40 pregnant migrant WLWH were conducted from June 2020-June 2021. Participants were recruited through purposive sampling at a public hospital in Johannesburg. A thematic approach was used to analyse interviews.

**Results:**

Forty interviews were conducted with 22 cross-border and 18 internal migrants. Women in cross-border migration patterns compared to interprovincial and intraregional mobility experienced barriers of documentation, language availability, mistreatment, education and counselling. Due to border closures, they were unable to receive ART interrupting adherence and relied on SMS reminders to adhere to ART during the pandemic. All 40 women struggled to understand the importance of adherence because of the lack of infrastructure to support social distancing protocols and to provide PMTCT education.

**Conclusions:**

COVID-19 amplified existing challenges for cross-border migrant women to utilise PMTCT services. Future pandemic preparedness should be addressed with differentiated service delivery including multi-month dispensing of ARVs, virtual educational care, and language-sensitive information, responsive to the needs of mobile women to alleviate the burden on the healthcare system.

## Background

The COVID-19 pandemic has presented challenges for South Africa (SA) to further eradicate HIV/AIDS and reach the UNAIDS 90–90-90 targets [1]. Although SA has the largest number of people enrolled on an ART programme, there has been an increased number of people living with HIV (PLWHIV), which has increased by 4.4 million from 2002 to 2021 [2]. A greater concern remains about the impact of COVID-19 on PLWHIV due to ‘immunosuppression’, ‘multi-morbidities’, and ‘higher mortality’ risks [3], especially for vulnerable populations including pregnant women living with HIV (WLWH).

Data suggest that only three months of HIV Prevention of Mother-to-Child Transmission (PMTCT) service disruption could result in new HIV infections spiking by as much as 53% for children up to 15 years old [4]. PMTCT typically entails women as healthcare users having access to services such as antenatal clinical care (ANC), HIV counselling and testing (HCT), antiretroviral treatment (ART), education on safe delivery and infant feeding, follow-up HIV testing at a 7-day postnatal visit, family planning, and lifelong ART [5,6]. PMTCT using ART is a proven, effective intervention and can reduce vertical HIV transmission to less than 2% [5,6].

A standardised approach remains challenging due to pre-existent structural and organisational factors that play into the provision of care [5,6]. In addition, the impact of the pandemic on HIV prevention for women is likely to be disproportionately high, as women bear the brunt of the HIV prevention service gaps [4].

Research shows that the COVID-19 pandemic interrupted HIV services, but the extent to which PMTCT access, treatment, and service delivery were impacted in SA remains unknown [2,7].

### COVID-19 impact: migration and WLWH

In March 2020, the World Health Organisation (WHO) declared COVID-19 a pandemic and SA restrictions resulted in a lockdown and international border closures that limited the movement of individuals [2,8]. As the spread of COVID-19 continues across SA, the most populous province of Gauteng consisting of 16 million people, of whom 13.7% are HIV-positive and a fourth of whom are migrant women in the reproductive ages of 15–49 years, have contributed to 1.2 million SARS-CoV-2 cases [2,7].

In 2020, the International Organisation of Migration (IOM) estimated that there were 4 million cross-border and internal migrants [2,9–11]. Cross-border migrants are defined as individuals who move from one country to another, and internal migrants are defined as individuals who transcend borders within a country. This includes an individual who moves within Gauteng but across the City of Johannesburg (CoJ) border in SA.

Statistics of SA suggests that approximately 60% of the migrant population is concentrated within the urban areas of SA, 32% are internal migrant women, and only 8% are cross-border migrants, many of whom are WLWH and who are highly mobile [2,9–11]. Highly mobile refers to an individual located within the peripheral and urban areas of the CoJ contributing to intraregional, interprovincial and/or cross-border migration patterns. Intraregional migration consists of individuals who move between the seven regional borders within Johannesburg, and interprovincial migration refers to individuals who move between the nine provinces of SA [2,9–11].

The impact of cross-border migration on healthcare systems during the COVID-19 pandemic is little understood but remains a highly political issue for SA as a recipient country [12]. Migration disrupts access to healthcare, posing significant risks for WLWH, including cross-border migrants, especially during lockdown and border closure periods [13]. Cross-border and interprovincial migrants are often unable to access healthcare services in SA due to the requirements of documentation. This has placed restrictions on their freedom of mobility and calls for governments’ own COVID-19 prevention strategies, both at provincial and national levels [13]. Migrant WLWH should be seen as a high-risk group and be advocated for when it comes to promoting respect and equal access to healthcare, especially for those without documentation [14].

After the establishment of the Border Management Act, the 2017 White Paper on International Migration had a vision that ‘South Africans must embrace internal migration for development while guarding sovereignty, peace, and security’ [14,15]. Despite these data and existing evidence on the diverse population movements within SA, the public healthcare system, on whom most of the population relies, has struggled to consider mobility or migration [13,14]. The SA constitution indicates the right to access healthcare for all who live in the country, and the nation’s health policy provides free primary healthcare access for all, with no mention of nationality and legal status. However, the interpretation of legislation remains less inclusive in its practice and the constitutional right to healthcare is often ignored [14,16,17].

Research suggests that mistreatment including xenophobic attitudes and practices by healthcare workers (HCWs) can impact provision and access to care for individuals based purely on their identity as a ‘non-national,’ also known as a non-South African citizen [17,18]. Studies provide examples of declining ‘non-nationals’ entrance into healthcare facilities and service providers placing ‘non-nationals’ in a longer queue [16–19]. ‘Non-nationals’ are often unable to receive the best care available because of the presumption of an increased burden they place on the SA public healthcare system [18–20].

Many cross-border migrant women do not have access to healthcare due to stigma, and are fearful of being asked for documentation and deportation [18–20]. The pandemic has added a layer to the pre-existent structural discrimination that has largely influenced health-seeking behaviors and social exclusion of both internal and cross-border migrant women within healthcare facilities [21]. Cross-border migrant women utilising PMTCT services face many of these barriers that disrupt their ability to adhere to medication and treatment for chronic diseases such as HIV, which requires long-term discipline [13,17,18,22].

An increased HIV disease burden is occurring across the country, and its relationship with migrants is complex and contested [15,23]. Migration is a continuous, repeated process rather than a single event, and therefore, the timing of migration in relation to past migration is significant; the first time an individual migrates, for example, leaving a family home, is very different from subsequent migrations and may be linked to different HIV risk exposure [23].

A migrant gap exists with policy and practices of PMTCT service provision and incorporation of highly mobile WLWH as users in SA, during the pandemic [2].

### Utilisation experiences of PMTCT

Although the PMTCT cascade represents a complex system of sequential, interdependent steps that WLWH navigate as healthcare users to receive appropriate care and treatment for themselves and their newborns, the National Department of Health’s PMTCT guidelines do not address the complexities of mobility and healthcare access for these women in high mobility contexts [2,5]. The vulnerability must be acknowledged when looking at risk factors of acquiring HIV both as a highly mobile migrant and as a woman [20,22]. WLWH and their interactions with the healthcare system may very well differentiate from each other due to different variables (e.g. valid documentation, HIV status, place of residence, and/or languages spoken) [13,20,22]. It is crucial in understanding that this process does not allude to one story but many different processes and experiences for the individual [23,24].

Studies on migration and health have focused mostly on defining migrants in the dichotomy of non-nationals versus nationals, where methodologies have used nationality and the ownership of citizenship and documentation to predetermine categories of migrants [23,24]. Research has shown that human behaviour commonly groups people into categories with the aim of making sense of the environment we live in [23]. However, the risk is that these ‘groups’, based on citizenship/documentation, become ‘single stories’ that give incomplete and simplistic understandings of the identities of others [24,25]. Instead, research to understand the influence of population movement on healthcare systems should consider the complexities of migration [25].

It is important to recognise that mobile populations may be categorised into more nuanced typologies with different compositional, contextual, and collective characteristics that may contribute to their healthcare experiences [14,23,24]. For example, typologies may include nationals who are non-national permanent residents, where an individuals’ place of birth is outside SA but they reside 5 or more years within SA, displaced individuals also known as someone who is forced to leave their home of habitual residence due to conflict but found safety internally in their own country of SA, and asylum seekers -defined as individuals who left their home country as a political refugee and is seeking asylum in SA [14,23]. Asylum seekers may have refugee status and are allowed to work in SA, and migrants with or without documentation such as no form of identification (ID), including a passport and/or ID card [14,23]. Research suggests that cross-border and internal migrants including interprovincial and intraregional sub-categories are relevant to the context of this research because of the vast movements that occur within the urban and peripheral areas of CoJ and Gauteng province [14,23]. However, by pre-determining typologies of migrants, it limits the ability to understand how migration and mobility patterns affect healthcare user experiences.

A literature gap exists, highlighting the need for a better understanding of different typologies of women and their experiences utilising healthcare in a high mobility context, the impacts of migrant status on their access to PMTCT healthcare, and their overall ability to adhere to lifelong care during the pandemic. This research aimed to qualitatively explore these experiences of different mobility typologies of migrant women utilising PMTCT services at a public hospital in CoJ and how belonging to a specific typology might have affected the healthcare received and, their overall utilisation experience during the COVID-19 pandemic.

## Methods

### Study site

**Figure 1.**
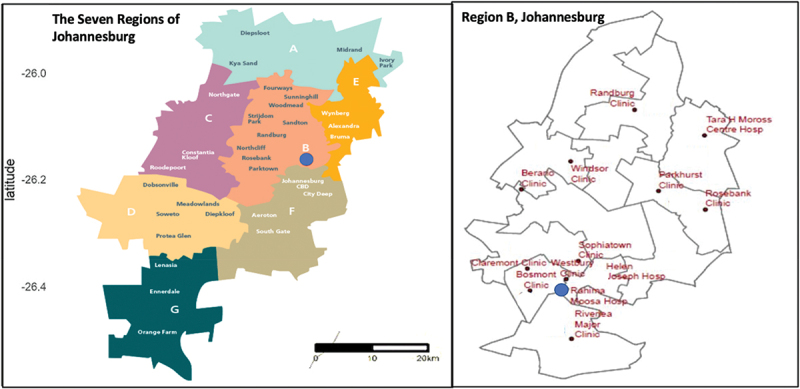
Regional Map, Region B, Rahima Moosa Mother and Child Hospital

Rahima Moosa Mother and Child Hospital (RMMCH) is a public hospital that provides PMTCT services in Coronationville and western Johannesburg catchment areas in Region B (Figure 1) [26,27]. Region B is often associated with more developed areas and suburbs, but this is not the case in Coronationville, where vast patterns of migration occur [26,27]. In the early 1990s, Coronationville experienced rapid urbanisation, resulting in the establishment of informal settlements [26,27]. Coronationville has a population of 7348 people, comprised of 2500 people, of whom 60.8% are females living in informal settlements [26,27]. Forty-four percent of its entire population is unemployed, of whom 68% are internal migrants [26,27].

In 2015, RMMCH was the first to implement PMTCT lifelong treatment [13]. RMMCH is a site associated with high mobility and different migration patterns in Gauteng, where many WLWH use services outside of their districts/catchment areas and it informs the conditions for applicability to the broader migrant population in SA. Approximately 25 clinics refer patients to the ANC, no matter their nationality or legal status, and 87% of women using the ANC and post-natal care (PNC) are recorded as non-SA citizens. It is a busy maternal healthcare environment where HCWs see more than 36,000 outpatients and more than 12,000 births annually [6]. RMMCH had adopted the national guidelines for PMTCT lifelong treatment and was in its monitoring stage, providing a platform to analyse different mobility typologies and migrant experiences in the programme during the pandemic.

### Study design

Data were collected for 13 months (June 2020-June 2021) during the COVID-19 pandemic. This qualitative study consisted of 40 semi-structured interviews where data saturation was reached [28]. Interviews uncovered WLWH and their experiences utilising PMTCT services at RMMCH. Typologies were used to specifically analyse different migrant WLWH and their movement patterns, referred to as *mobility typologies* [14]. The different mobility typologies that surfaced and how belonging to a specific typology may have affected the healthcare received were examined. To avoid narrow conceptualisations of migrants, the wide range of typologies occurred was identified, where different classifications assigned to the participants based on socio-demographics, clinical characteristics, and their overall experiences shared in the utilisation of PMTCT services were used.

### Study recruitment

Study participants were recruited from the ANC. Participants had to be of the reproductive age of 18 years or older; HIV-positive; you cannot be pregnant and newly delivered, or in post-natal follow-up. WLWH had to be enrolled in the PMTCT service and already initiated onto ART at RMMCH to be eligible for inclusion. Participants were not excluded based on language, and a translator was used on-site. WLWH were initially identified and asked by the nurse if they would like to participate in the study on a voluntary basis when conducting their follow-up assessments. If the individual woman agreed, they were referred to the research assistant/translator and PI in a private room within the ANC. The names and contact details of those who agreed were then shared with the research team. Participants were provided the study information. Approximately 35 women were approached but declined, mainly due to the peak of the second wave of COVID-19. These individuals were concerned with staying longer at the hospital because of the risk of contracting SARS-CoV-2.

### Data collection and management

A purposive sample approach was used to recruit patient participants on PMTCT at RMMCH [28]. Due to the hospital regulations and the implementation of COVID-19 protocols and procedures, data collection was delayed because of the lack of space to follow social distancing protocols in the hospital. A tent was set up outside the ANC to provide a ventilated area for patients to sit and wait until their number was called to enter the facility to receive their care. The recruitment of participants had to be adjusted for the first 2 months because potential participants would have to return outside or move to another area of the hospital to complete the interview process to maintain confidentiality.

Those who provided consent then completed an in-depth interview (IDI) in a private room. This research on sensitive migrant/WLWH participants ensured the protection of human subjects, and a distress protocol was followed for participants who became distressed by the nature of telling their story.

An interview guide was informed by the conceptual framework and was used to collect data on 1) migrant status, 2) geography, 3) mobility history/temporality, 4) motivations/casual classifications, 5) socio-demographics, and 6) users clinical characteristics [25,28–31].

Interviews were audio-recorded and conducted in Tswana, Sotho, Zulu and Xhosa, Shona, Ndebele, Tumbuku, Changana, Chichewa, Afrikaans, and English and then were transcribed verbatim by one research assistant/translator and 1–2 interviewer(s). One counsellor who spoke Chichewa fluently only assisted the translator in Chichewa interviews if the participant struggled to communicate at times.

To ensure participant confidentiality and anonymity, all results were aggregated and assigned a unique identification number that only the PI had access to, to ensure that participant responses could not be linked to them [28].

Demographic information, observations of participant behaviour, and journal memos were entered into NVivo 12.0 (QSR International Pty Ltd., 2018). To explore any preconceived notions from the research team, interviews used reflective journaling as an opportunity to engage with the data collected and daily journal entries more critically to enhance authenticity and learn from past experiences of the research process and findings [28,32]. Memos and journaling were used to maintain a process log, capture any observations, and maintain a record of analytic decisions throughout data collection and analysis between the research team [28,32].

### Conceptual framework for exploring utilisation of PMTCT healthcare services

**Figure 2.**
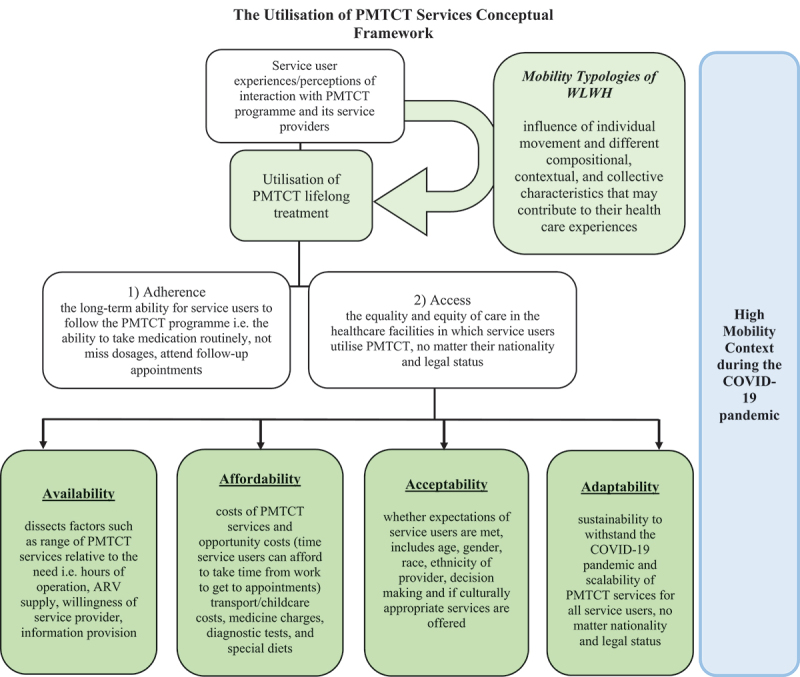
The Utilisation of Prevention of Mother-to-Child Transmission (PMTCT) Services Conceptual Framework

The Utilisation of PMTCT Healthcare Services Conceptual Framework (Figure 2) was developed to provide guidance in exploring utilisation experiences of PMTCT and migration in a high mobility context [6,28–31].

McIntyre et al.’s Accessibility framework informed this framework, highlighting that by using ‘access’ as a concept, this would not include those migrants who have not accessed PMTCT services for comparison [6,16,29]. Utilisation is defined as the WLWH experiences/perceptions of their interactions with the PMTCT programme components and to the service providers within a high mobility context [16,29]. The framework was applied to explore accessibility and adherence barriers and facilitators in relation to PMTCT services and how the individual story of migration, and the mobility typology, has impacted their overall healthcare experience. The concept of utilisation is made up of 1) *adherence* issues to the programme and 2) *access* composed of four dimensions: *availability*, *affordability*, *acceptability*, and *adaptability* [6,16,29]. Adherence is conceptualised as the long-term ability of these different women to follow the PMTCT programme including taking medication routinely, not missing dosages and attending follow-up appointments. Accessibility refers to the equality and equity of PMTCT care in the healthcare facilities these women utilise, no matter their nationality and legal status [6,16,29]. Availability dissects the factors such as the range of maternal healthcare and PMTCT services relative to the need. This includes the hours of operation, ARV supply, and willingness of service provider to provide information in a demanding clinical environment [6,16,29]. Affordability refers to both the financial costs, which include transport, medicine charges, diagnostic tests, and childcare costs, and the opportunity costs, which refer to the time a woman can afford to take time from work to get to the hospital to receive PMTCT services [6,16,29]. Acceptability refers to whether the expectations of the women are met, including age, gender, race, ethnicity of provider, decision-making, and whether culturally appropriate services (e.g. multi-lingual counselling) are offered [6,16,29]. Acceptability also includes the woman’s non-medical expectations of HCWs’ treatment, dignity, and respect of their culture [6,16,29]. Adaptability refers to the sustainability and scalability of the PMTCT programme for all women, no matter their nationality and legal status [6,16,29].

Analysis

Data were compared to draw similarities and differences amongst participants [28,30–32]. First, the dimensions of adherence and access were used to dissect and draw relationships to each of the participants’ experiences. The typologies of migrants that surfaced were grouped based on their experiences that were similar or different. Second, the data on 1) migrant status, 2) geography, 3) mobility history/temporality, 4) motivations/casual classifications, 5) socio-demographics, and 6) users’ clinical characteristics were analysed to help guide and categorise the different typologies that arose. Typologies are different classifications assigned to the participants based on the six categorical variables (Table 1) that provided a profile for the participant to draw further relations with the participants’ utilisation experiences of PMTCT services [25].

**Table 1.** Examples of the factors/variables involved in the development of migrant typologies.

Categorical Area	Factors/Variables
Migrant Status	Nationality, documentation status, asylum seeker, refugee status, displaced persons, job-seeker, migrant worker
Geography	Rural to urban migrants, intra-urban migration, inter-regional migration, internal migration, cross-border migration, return migration
Temporality	Time in transit, frequency of trips (weekly/monthly commuting), seasonal migration, contractual migration, short-term migration, location of facilities visited
Socio-demographic status	Age of patient, family structure, household income, employment status, education level
Motivations/causal classifications	Job seeking, family reunification, asylum seeking, refugee resettlement, delivery of baby, studies
Clinical characteristics	Year of HIV diagnosis, attendance rate/number of healthcare facility visits, duration on PMTCT lifelong treatment, date of delivery, outcome of baby (HIV status)

A thematic analysis was used to analyse the semi-structured interview transcripts to examine and identify common themes, topics, ideas, and patterns of meaning that were repetitive [28,30–32]. Interviews were approximately 25–35 minutes.

The process of analysis began with open coding directly from the data [28,30–32]. Codes were grouped into sub-themes that emerged from the data and then organised into the themes of adherence and the four dimensions of accessibility. Two researchers in the team coded individually. The consensus was reached at the third version of the coding scheme among three researchers in the team. The categories were reduced to identify key themes. Any commonalities and differences between utilisation experiences across WLWH were noted. Triangulation of data was used to ensure that different experiences of study participants were presented [28,30–32].

Descriptive statistics were reported for the demographics of the participants, including frequencies and proportions for categorical variables using Stata 14 [28,30–32].

## Results

### Demographics and characteristics

Forty interviews were conducted with WLWH, between 20 and 42 years old. Participants consisted of 22 cross-border and 18 internal migrants (11 interprovincial and 7 intraregional migrants). Of the 40 participants, 17 were non-national permanent residents, 2 sought asylum, 2 were without an address and displaced, and 4 women were without a valid passport or ID card (Table 2). The duration on ART ranged from 1 week to 20 years for participants, many of whom initiated ART at the clinic with the same-day initiation (SDI) policy. The duration of pregnancy ranged from 6 weeks to 8.5 months.

**Table 2.** Demographics of participants.

Variable (number of responses)	Sub-category	Frequency
Mobility Typologies (40)	Cross-border	22
	Internal	18
	Interprovincial	11
	Intraregional	7
	Non-national permanent residents	17
	Asylum seeker	2
	Displaced individuals	2
	Individuals without form of identification (passport/ID card)	4
Age years (40)	18–24 25-35	6 23
	36–45	11
Highest level of education (40)	Primary to Grade 7	23
	Grade 8–10	3
	Grade 11	8
	Matric/Grade 12	3
	Higher education	2
	Certificate programme	1
Occupation (40)	Employed	14
	Self-employed	3
	Temporary employment	2
	Unemployed	20
	Missing	1
Marital status (40)	Married	14
	Engaged	1
	Single	5
	Co-dependent/living with partner	19
	Missing	1
Number of dependents (not including current pregnancy) (40)	0	11
	1	13
	2	11
	3	4
	>3	1
Place of birth (40)	Other province in SA	11
	In Johannesburg	4
	In Gauteng but outside	3
	Johannesburg	19
	Zimbabwe	2
	Malawi	1
	Mozambique	
Residing (40)	With family in Johannesburg	4
	With partner in Johannesburg	33
	Single household	3

Most women (22) were from another country, including Zimbabwe, Malawi, and Mozambique, and spoke a beginner level of English. Eighteen of these participants preferred to conduct the interview in Shona, Ndebele, Sotho, and Chichewa. Interprovincial and intraregional participants primarily spoke Zulu. Three women presented with high-risk complications including blood pressure, previous miscarriages, and/or ectopic pregnancy. The relationship a participant had with the ANC differentiated, ranging from first visits, follow-ups, and delivering a second/third child at RMMCH.

The Utilisation of PMTCT framework included (1) adherence and (2) the four dimensions of access: availability, affordability, acceptability and adaptability, which were used to organise subthemes derived from the IDIs (Table 3).

**Table 3.** Themes and subthemes

	Themes	Subthemes
Adherence	Facilitators and barriers for adherence to PMTCT services	Solely for health of the baby Treatment interruption: Relocation and side effects Fear
Access		
Availability	Clinical environment for service users	Space PMTCT education and counselling
Affordability	Financial constraints and opportunity costs to receive PMTCT care	Job loss in lockdown Documentation
Acceptability	Interpersonal interactions (between healthcare users and PMTCT service providers)	Mistreatment
Adaptability	Services for different movement patterns	Same day initiation (SDI) Differentiated service delivery (DSD) -Individualised approach

## Adherence: facilitators and barriers for adherence to PMTCT services

The adherence theme was composed of the subthemes, ‘solely for health of the baby’, ‘treatment interruption: relocation and side effects’, and ‘fear’.

### Solely for health of the baby

All participants, no matter their nationality, were asked, *‘why do you take your treatment?’* All women responded that their main driving factor for adhering to ART was their concern solely over the outcome of their baby. Nothing else mattered in comparison to the babies’ health because they had lived with HIV so far and survived challenging life circumstances.

Most interprovincial migrants did not know about the PMTCT programme in great detail and understood it as simply taking ARVs, to allow them to breastfeed their baby. A 26-year-old interprovincial migrant, who resided for nine years in Johannesburg, understood it as, *‘If you’re HIV positive, you have to take medication and do exclusive feeding. You shouldn’t do breastfeeding because the child shouldn’t be exposed to cracks. You are to give the child medication for a recommended period of at least six months’* [IDI 1].

A 42-year-old interprovincial migrant with primary-level education emphasised the need for practical education from adherence counsellors to become better informed that PMTCT went beyond just taking pills,

*‘They told me that I have four options, but they didn’t tell me practically what that looked like … I need* [to] *not keep my struggle a secret knowing that somebody might help me if only I open up […] I’m told that my body needs to acclimatise to taking thepills’* [IDI 21].

Geographic mobility and COVID-19 seemed to have a greater impact on adherence for cross-border migrant women. They were concerned with the risk of pregnancy complications because of the restrictions to enter borders and receive care during the lockdown.

Cross-border migrants who had previous births were aware of their HIV status and adhered to ARVs. However, most felt they were left in the dark, a Zimbabwean woman who travelled to and from SA for the past 6 years stated,

*“You know when they do something, they just write on the paper. You go to the doctor and they write* [something] *down and when I get out, I then just see that they wrote something, but I don’t know what it means. I want to know with these tablets that I am taking if the viral load going up or going down”* [IDI 28].

Interestingly, very few South African nationals who spoke local languages of Zulu and Sotho understood the high risk of the new-borns exposure in comparison with cross-border migrants who received adherence counselling in another country outside SA. This indicated that more work needed to be done with the education component of the programme and supplying ARVs is not enough.

### Treatment interruption: relocation and side effects

The commonest reasons for interruption of ART for cross-border migrants were relocation, running out of ARVs, and the adverse side effects of ARVs. A shy 20-year-old from Zimbabwe who arrived a year ago found out about her status on the same day as her pregnancy. She expressed her concerns about running out of medication, *‘I am five months pregnant now. I arrived in Johannesburg and live with my boyfriend […] I have only been on treatment for four months and I just know I have to have enough to take mine all the time and* [I] *worry’* [IDI 22].

When compared to SA nationals, cross-border migrants reported more difficulty in understanding ARV side effects and medication dosages. A single 30-year-old from Zimbabwe on ART for 36 weeks explained, ‘*The one in a blue bottle doesn’t make me sleep, it makes me have hallucinations. Sometimes when I sleep, I’ll be having nightmares but I think it’s because of the medication’* [IDI 40]. A non-national permanent resident from Zimbabwe, married with two dependents in Zimbabwe, explained that she only understood dosages by speaking to a peer in her community of Zimbabwe, *‘They* [HCWs] *just write and then I* *take it to the pharmacy […] This lady told me that I* *have to drink them at night before I* *sleep. Then I* *ask*[ed],*how am I* *supposed to do that? She said, that’s how it’s supposed to be, you take them at 8 o’clock and then go to bed’* [IDI 29]. She was able to identify collectively with another WLWH from her country of origin and better understand the importance of adherence.

Cross-border migrants also relied on short message service (SMS) reminders and in mobile applications to improve their adherence to PMTCT care during COVID-19, ‘*On the phone, they put this app “MomConnect,” which reminds us of if we have any hospital visits and how you should take care of yourself when the child is about to come’* [IDI 32].

### Fear

Interprovincial migrants felt fear surrounding them in the healthcare environment. A 25-year-old from Mpumalanga explained,

*‘There are healthcare spaces where we are not permitted to enter because of the fear around COVID-19. I’m actually from a COVID-19 ward […] I was discharged because my results said that I was negative. People are ill in that ward, some are pregnant […]They don’t mix them, the ones that are positive’* [IDI 6].

Many interprovincial migrants interrupted their adherence to ART and appointments because of their ‘fear’ of contracting COVID-19 when picking up their medication from the ANC. Fear was amplified by cross-border migrants receiving care. Specifically, these women expressed fear of contracting COVID-19 because of their distrust in the ability of the hospital to follow regulations. A non-national permanent resident from Zimbabwe, married with six dependents, expressed her fear towards HCWs that were not following sanitary protocol measures when collecting her ARVs,

*“To be honest, I’m not comfortable because I am checking for this Coronavirus when I’m coming in. They say I must be checked* [and] *put on a mask. I follow those instructions because I need the service from the hospital. But once I get in, I’ll find some of the staff who are not following those regulations […] They* [nurses] *were not even changing the linen. If someone just comes out, they will just take another patient in […] to me I feel like I’m in a red-hot zone because I don’t know how safe I am. It* [COVID-19] *doesn’t affect other people and selects others”* [IDI 34].

She referred to being in a ‘red-hot zone’ as alarming and assessed these implications as a high risk for her to contract SARS-CoV-2, in what was supposed to be a safe hospital environment where precautionary measures are taken to provide the best possible care.

## Availability: clinical environment for service users

Participants discussed the healthcare environment during COVID-19 including 'space' and 'PMTCT education and counselling'.

### Space

There was a lack of consultation rooms because of social distancing and changes made to the hospital regulations. An intraregional migrant from CoJ was turned away from care,

*I gave them a* *call about my problem, and they replied by telling me that they don’t have sufficient consultation rooms and that’s caused this unfortunate situation. There’s nothing I* *can do, it means whenever I* *have to go that side, I’ll find that place as it is. Nothing is going to change [IDI 18].*

Intraregional and interprovincial migrants were turned away from care and were unable to complete their appointments at local clinics because of a lack of equipment and human resources to do their follow-up testing during COVID-19. A 36-year-old interprovincial migrant from KwaZulu-Natal expressed her frustration, *‘clinics are small in size and do not have sufficient equipment and don’t have doctors to cater for bigger needs’* [IDI 7].

Sanitary space was the main concern expressed by cross-border participants because if they were to contract COVID-19, the consequences would be more severe as a ‘foreigner who could not be with their child’ as the primary caregiver in Johannesburg.

Women who tested positive for COVID-19 were admitted to RMMCH if they have a high-risk complication,

*I had Coronavirus. I* *was admitted for two weeks due to my high blood pressure […] they told me to continue getting medical [treatment] here. I* *think that’s where many people with weak immune systems lose the battle against COVID-19. That’s why you can’t breathe because it chokes you. I’m pregnant and throwing up. I* *stay alone. I* *spent two weeks here and went to self-isolate for another two weeks [IDI* *1].*

Many cross-border, interprovincial, and displaced migrants were completely alone in Johannesburg and needed support, while their partner is working elsewhere or their families are back ‘home’. *Home* was often associated with the location of their loved ones. Therefore, having a space and medical equipment available, which followed social distancing and sanitation guidelines, remained a crucial need for cross-border and displaced migrants- who have nowhere else to go. They depend heavily on the SA healthcare system to be able to withstand any pregnancy complications during COVID-19.

### PMTCT education and counselling

All women, no matter their mobility history/temporality, suggested that the educational component of PMTCT could be improved. Many interprovincial and intraregional migrants were referred to RMMCH from clinics where they received most (if any) counselling and communicated that long waiting times could be taken as an opportunity to help educate women.

*“The experience here is good in terms of checking the child since they have free sonars […] but education-wise they don’t teach you anything. In Mpumalanga, when they give you medication like chronic tablets, they teach you how you should take it. Mind you a first-time pregnant woman will make mistakes because she doesn’t know anything relatedto her medication. I wish that while we’re waiting for the doctors to call us […] there may be people who are designated to educate us about this programme, doing presentations. They should do it the whole day. Even if they do a repetition, it shouldn’t be a problem because there are always new patients who come here”* [IDI 1].

Interprovincial migrants communicated that continuous education and knowledge sharing are ways to build their self-capacity and be able to prevent HIV transmission in their communities, *‘Yes, I would like to know more so that I can inform another woman in the same situation as mine*’ [IDI 10].

Some internal migrants received more information outside Johannesburg, *‘I once did an auxiliary programme, I first heard about it there. I also heard about it during my first pregnancy at the clinic in Mpumalanga. Here, they never told me anything about this programme’* [IDI 19]. Emphasis was also placed on the distribution of PMTCT education to intraregional rural communities for those who do not have the same modes of availability as others who live in the city. A mother who arrived in CoJ seventeen years ago, and spent 5 months on ART, conveyed the importance of knowledge translation with COVID-19 and being immunocompromised,

*[…] because a* *lot of people who live in the rural areas are still behind in terms of access to information. Those living in Gauteng are better off because of some knowledge they receive from watching television. On the other hand, people living in rural areas do not have TVs, or radios, and do not have people who sit them down and educate them about the reality of this virus. A* *lot of people die out of ignorance [IDI* *7].*

Due to border closures, cross-border migrants seemed to have more difficulty in navigating the healthcare information received from both their country of origin and CoJ, *‘When I had my first child I was scared. I didn’t breastfeed so I want to know how I’m going to cope. Is it high risk or? I want to know because last time I didn’t ask’* [IDI 30]. These WLWH needed to be equipped particularly with information on breastfeeding, which was a reoccurring topic. Breastfeeding created confusion with mixed messages received from different information sources such as healthcare professionals, media, and social networks.

However, two asylum seekers arrived in CoJ a few years ago and expressed a difference in opinion. They found that the information on PMTCT was very sufficient in comparison to the counselling they had received elsewhere, *‘The information is 100% because even my first child is negative’* [IDI 16]. It is important to recognise that providing information can empower women in different life circumstances. Overall, PMTCT education and counselling were needed as a crucial component of PMTCT to support mothers of all journeys.

## Affordability: financial constraints and opportunity costs to receive PMTCT care

Participants expressed financial constraints and opportunity costs including ‘job loss in lockdown’ and ‘documentation’ to receive care.

### Job loss in lockdown

Interprovincial and intraregional migrants could not endure transport costs to the hospital because of losing their jobs during the pandemic. There were not enough taxis during lockdown for intraregional migrants to get to RMMCH, *‘this place is far given the amount of money I pay to come here. I take two taxis for a single trip’* [IDI 11].

When investigating why some internal migrants migrated to CoJ, particular reasons included, ‘to give birth’, ‘receive better healthcare’ [IDI 34], and ‘have better work opportunities’ to help support their families [IDI 20].

A self-employed woman from Zimbabwe explained, ‘*I came to SA to look for a job. My parents passed away when I was young so I grew up with my grandmother. I finished school at the age of 16 so I had to look for a way to survive’* [IDI 2]. Predominantly, cross-border migrants’ main reason for moving to Johannesburg was employment opportunities.

Cross-border migrants significantly suffered from border closures. A Malawian participant and her boyfriend both lost their jobs and were ‘*left without an income due to lockdown’* [IDI 8]. The emotional toll was evident with financial stress for cross-border migrants. A displaced Zimbabwean migrant shared her very painful experience coming to Johannesburg to live with her sister, who was abusive and forced her to become a sex worker to put food on the table. She was distressed and expressed her inability to travel back to Malawi because of COVID-19,

*At first I* *felt ok, and everything was fine before I* *got raped. After that, I* *felt life was a* *struggle. I* *wished I* *could go back home. At the time, I* *didn’t have the means to go back home. Later when I* *got some means, I* *travelled back home. I* *then came back to SA* *to try to make a* *living [IDI 17].*

Although both internal and cross-border migrants needed financial support, cross-border migrants had to endure more opportunity costs to seek healthcare with border restrictions and very limited family support.

### Documentation

Opportunity costs to use PMTCT services were amplified specifically for cross-border WLWH, where they could not attend appointments because of documentation issues. Cross-border and displaced migrants from Zimbabwe and Malawi expressed how the documentation was a major barrier to utilising services during the pandemic,

*It was very hard. We would travel on one bus and then step off to take another one. This occurred several times. Before we arrived at the border, we were arrested. When we arrived at the border we got arrested again. Even when we had passed through the border we still got arrested […] My sister had identification, but I* *didn’t. They thought she had kidnapped me [IDI 17].*

Documentation served as proof of a ‘legal status’ for cross-border migrants to enter SA to access healthcare and often crippled these WLWH who found themselves under difficult life circumstances. One woman from Malawi stated she was arrested multiple times and even stuck with an expired passport during the lockdown. She had to prove her story to staff constantly,

They [nurses] said I don’t have a permit and then I showed them my passport that I came here as [a] visitor then the lockdown closed me [on] this side of SA. Then my passport expired on the 31st of March. Then the second time, I ended up going to the nurse in charge and showed her my passport and I said if you check my pregnancy, it’s for July and my passport expired in March when the pandemic started … [IDI 34].

Implementation of the COVID-19 appointment booking system ultimately caused chaos for cross-border and interprovincial migrants who could not afford to take time off work (whether working in a different country/province of origin) to travel to the clinic because of border restrictions. A single 33-year-old Zimbabwean woman had contracted COVID-19 from a client at work and was stuck during the lockdown. She was booked for delivery and was trying to use an affidavit. She expressed her difficulty in attending her appointment because of her passport being robbed,

It wasn’t safe to pass through the passages. The phone they can take but the passport- because now when you are renewing, they must see the old one, that was my fear. It won’t be a long process, I just won’t get another one, that’s the end of the story because they want to see it. I can’t [use] my word of mouth, nor a photocopy, only the original [IDI 33].

## Acceptability: interpersonal interactions

Participants discussed ‘mistreatment’ regarding their interactions with HCWs during COVID-19.

### Mistreatment

It was common for SA nationals to report they were never denied treatment at RMMCH. More SA nationals from Gauteng expressed they were comfortable when speaking with nurses, ‘*because when there’s something I don’t understand, I do ask questions and receive feedback*’ [IDI 14]. A 39-year-old intraregional migrant, employed, who regularly attended the hospital since relocating to Johannesburg, and was pregnant with her second child, thought otherwise. She stated there was a lot of ‘*negligence’* in the labour ward and ‘*the environment was not conducive. The treatment was the issue. Some patients were in pain, yet no one was attending to them. One of the patients gave birth in a toilet’* [IDI 18].

Interprovincial migrants expressed that treatment differentiated based on whom they interacted with and that the effort had to be made by both the provider and the user, *‘The treatment you receive from the nurses is dependent on how you as a patient address them and how they’re feeling at that time they assist you. They don’t segregate in terms of background and status. For instance, […] when another person comes to talk to me, my reaction will all depend on my mood at the time. I’ve never had problems of segregation here’* [IDI 7].

In contrast to SA nationals, many cross-border migrants spoke about their increased frustrations being separated from ‘locals’ and waiting in longer queues. Queues became more difficult with COVID-19 guidelines, ‘*I have a problem with long queues. Look at me now, I’m number forty-one despite the fact that I arrived at 6:30 a.m. We are a great number. It is cold, and we’re in danger of contracting COVID-19’* [IDI 9].

Cross-border migrants reported poor behaviour and attitudes of nurses including being shouted at and called names such as ‘defaulter’ or ‘foreigner’ and were unaware of whom they could even speak/report to,

Some of the nurses, like when we go to the labour wards. Yoh! It’s so terrible- but not all of them. I can’t assume why they do that. Do they do that to the foreigners only or do they do that to everyone? Or they’re just like that? They just don’t have time. They just don’t have the care. Because sometimes when you’re in labour pain, you can’t control yourself and that thing- you have no control over, but some [nurses] take it like it’s their high time to abuse someone, to abuse patients [IDI 34].

Cross-border migrants, who newly arrived in SA, expressed that poor communication from nurses led to their distrust in the healthcare system. A non-national permanent resident from Zimbabwe reflected on how she feared having a caesarean section when she first arrived in SA ten years ago,

I was scared because this was my first time, I was still young. I was also referred there from the clinic to go and do a caesarean birth […] I spent a week in Johannesburg. I was crying every day and thinking of running away […] I was being told that if I would have a caesarean birth, I would die. There are so many things that we are told about and hear, […] even the sisters knew me as this girl who cries a lot and just wished that I would even go back home [IDI 27].

Struggling with integration into a new space within the city was a common experience by many, whether from a different country, province, or moving from a rural area within Gauteng. Cross-border migrants had feelings of abandonment, wanting to belong, to be a part of the community, and not be left behind because of their HIV status or being a ‘foreigner’.

## Adaptability: services for different movement patterns

Participants were asked about areas to improve and better support their migration journey. Sub-themes included ‘SDI’ and ‘differentiated service delivery’ (DSD).

### Same day initiation (SDI)

Women waited outside the counselling rooms and interacted with each other, laughing as they expressed that they did not see the point with testing when there was insufficient time for counselling to be conducted all in one day. A 35-year-old interprovincial migrant, on ART for 3 years stated, ‘*The only problem is that they don’t give you all the information you need. They leave you ignorant’* [IDI 9].

Many participants from different mobility journeys only knew they have to take ARVs but lacked the answers as to why. The simple concise messaging that was provided lacked stressing the importance of long-term adherence post-delivery to patients.

There was a lack of ensuring confidentiality at all times during SDI, especially for cross-border migrants who remained the most vulnerable, without proper counselling or good education to conduct research on their own about HIV/PMTCT. A single and unemployed woman from Zimbabwe expressed, *‘I desire to know about this programme but they don’t have time to do counselling’* [IDI 12]. Time was a major factor for SDI to ensure that these vulnerable women had the information they need to really benefit from the programme.

Indirect disclosure occurred with SDI because of clinic flow, the constraints of space, the limited time spent with the counsellor, and the different queues that segregated women by their HIV status. Cross-border migrants compared RMMCH to other clinics they used, *‘There is a difference. In Zimbabwe, people are segregated along the lines of languages. Some speak Shona, others speak Ndebele’* [IDI 3]. The language remained a large barrier for many cross-border participants (18) who expressed they were not as comfortable speaking in English.

### DSD: individualised approach

Participants spoke about different ways to help alleviate the challenges with their use of PMTCT services during COVID-19. Interprovincial and intraregional migrants recommended outreach services would be beneficial during the pandemic. A 35-year-old who had a stillborn stated, *‘It would be a good idea for nurses to visit patients at their homes for check-ups to curb the long queues and the spread of the virus. We all have different challenges’* [IDI 13].

One common challenge expressed by participants was the need to access ART for a longer duration of time, also known as multi-month dispensing (MMD).

All women were referred from local clinics to RMMCH because of high-risk complications and nurses required a referral letter. Two cross-border participants were waiting on permanent residency and struggled without letters or asylum-seeking papers to utilise healthcare in Gauteng.

RMMCH remained reputable amongst different mobility typologies utilising PMTCT services, who expressed they were not turned away like other clinics when they did not have documents or speak the local languages.

A woman born in Mozambique, who spoke little English, arrived from Maputo a few months ago. She commented that she found RMMCH to have better services to offer than back home, *‘Because I didn’t get tested in Mozambique. I really don’t know what they are doing there. RMMCH helped me choose the time for drinking the pills’* [IDI 15]. This presented a contrasting case, where a cross-border migrant found the PMTCT information received more useful than her country of origin. The fact that translation services were available to her through a counsellor at the clinic who spoke Changana and provided information in a language she could understand proved it was beneficial to her as a new arrival in CoJ. This indicated two crucial elements, a willing service provider to take the time to provide information and translation services for individuals who do not speak any of the local languages, to better understand and benefit from the information provided.

## Discussion

This research qualitatively explored the individual experiences of WLWH from different mobility typologies, using public healthcare services in the high mobility context in CoJ. It is a contribution to knowledge by both identifying mobility typologies including cross-border, asylum seekers, displaced individuals, interprovincial, intraregional, and non-national permanent residents of women who surfaced from the data and the similarities and differences between their utilisation experiences of PMTCT services. This study extended literature beyond predefined labels for migrants that tend to limit descriptions to the dichotomy of nationals versus non-nationals and allowed for more tailored strategies responsive to the service needs of highly mobile women [25].

Most women (57%) had a primary to grade 7 level education, were between 25 and 35 years old, had one or two dependents, were unemployed, and were co-dependent. Fifteen women had taken ART for less than one year, 12 of whom were cross-border migrants, 4–8 months pregnant, and had initiated ART for the first time at RMMCH. This remains an alarming concern for many cross-border migrants who are newly diagnosed only when accessing available PMTCT care at a state hospital for the first time.

The utilisation of HIV/PMTCT was restricted during the pandemic due to lockdown periods that limited the capacity and movement of migrants [33]. Findings contributed to an explicit narrative to help address the challenges with the HIV health system response for these mobility typologies and consider the individualised experiences of these women when developing strategies to prevent future interruption of PMTCT care [34].

The following was derived from the conceptual framework, which was valuable in providing a better understanding of the adversities faced by different mobility typologies in both adherence and the dimensions of access, to help determine a way forward to address these challenges.

### Cross-border migrants

Geographic mobility and COVID-19 seemed to have a greater impact on adherence for cross-border migrant WLWH because of the lockdown restrictions. The commonest reasons for interruption of ART were relocation, running out of ARVs, and the side effects of ARVs. There was a heavy reliance on SMS reminders, which could strengthen retention to the programme.

The dimension of availability indicated that although all women, no matter their nationality, struggled to understand the importance of adherence to ART post-delivery, cross-border migrants had significant challenges with navigating PMTCT information received because of poor communication by HCWs [6]. It is important to target key messages on breastfeeding while on ART in various languages and at a primary education level that many in the catchment area will understand. Health talks should be conducted both in-person and virtually, taking advantage of long waiting times to provide PMTCT education and adherence counselling. This will build the self-capacity of patients, through the empowerment of information, made available in different languages.

Affordability highlighted that it was common for these women to be the primary caregiver without a support system physically present and undergo financial burden during the lockdown. Documentation remained a significant opportunity cost for asylum seekers and displaced individuals. They could not attend appointments because of difficult life circumstances including job loss and stolen/lost documentation. DSD could combat this by offering a unified pathway for migrants and healthcare providers across the care continuum [35–37]. DSD can provide a verifiable standardisation of care and a lifelong longitudinal digital patient health record for cross-border migrants and different mobility typologies [35–37].

The dimension of acceptability revealed more fear and reluctance of cross-border migrants to utilise services because of their distrust in the ability of the hospital to follow regulations. Briefing sessions should be executed weekly/monthly (depending on the frequency of changes that occur) to better inform HCWs on the updated protocol and procedures during the pandemic.

Mistreatment and discrimination were existent towards these individuals from some HCWs. They were often placed in longer queues, segregated by language, called names, and lacked confidentiality from staff [16]. Language should not be a gateway or equivalent to whether the patient is ‘enough’ to receive a service [37,38]. Service providers should be trained on language sensitivity and behaviour by conducting value clarification workshops. HCWs should be provided psychosocial support to assist in managing poor behaviours and xenophobic attitudes towards cross-border migrants [14–19]. It is important for providers to better understand that an individual’s place of birth or where they reside does not solely define them.

### Interprovincial migrants

Availability highlighted that some interprovincial migrants craved practical education when receiving adherence counselling, and demonstrations should be conducted by HCWs.

Most experienced fair treatment depending on the HCWs they interacted with in general. However, there was not enough time to have thorough counselling conducted and indirect disclosure occurred due to segregating patients by HIV status [6]. A rotation schedule should be implemented to allocate time for counsellors. Precautionary measures need to be taken by management to ensure that privacy is available for patients with any space made available.

Acceptability revealed that some individuals were turned away from clinics and unable to complete appointments because of the lack of human and structural resources. Research has indicated that the impact of the COVID-19 lockdown significantly reduced ART services by 46% in 65 South African primary care clinics [35,39]

Adaptability indicated the provision of PMTCT during COVID-19 should include bundling services in the same visit and providing services outside the facilities including at-home visits or pop-up clinics. This could reduce the risk of exposure to COVID-19 where vulnerabilities are amplified for individuals who are often turned away at facilities [35,36,37,39,38].

### Intraregional migrants

Challenges stood out in the availability of space with COVID-19 protocols and the resources to complete patient appointments. The healthcare system should enable migrants to access the chosen services through multiple channels and touch points with as little friction as possible to reduce the burden on patients and healthcare systems [35]. Flexible ART collection and pick-up point dates/times strongly influence long-term engagement in HIV care [35,36,37,39,38].

Affordability revealed more intraregional migrants, and non-national permanent residents appeared to gain access to and utilise services easily when compared to other WLWH crossing international borders, asylum seekers, or migrants without documentation. However, most intraregional migrants were unable to take time off work or have transport to the hospital because of the limited number of taxis during lockdown [16]. The overburdened clinical environment and COVID-19 impacted the availability of services, especially WLWH in rural areas with limited access to the clinic.

### Non-national permanent residents

Findings demonstrated that some non-national participants can have permanent residency in SA and still strongly identify with different compositional, contextual, and collective characteristics which are fluid due to their mobility. There is a ‘biosociality’ that ‘arises from belonging and identifying with a biological condition and shared therapeutic practice’ [40]. By looking at the unique journey with mobility, migration, and HIV at an individual level, channels of support should be embedded into the programme delivery [39,40].

Struggling with integration into a new space within the city was a common experience by many, whether from a different country, province, or rural area. For service responsiveness to better integrate PMTCT care on a larger scale, adherence support groups should be available in different languages where mothers can connect with a community they identify with [35,36,37,39,38].

Migration and health are not static, and the policy and programme actors need to take action this research and review the healthcare system in SA. The healthcare accessibility rights of these individuals should be advocated for by key stakeholders to provide the best care possible and not discriminate against or exclude migrants, especially highly mobile WLWH who remain vulnerable and are already at a disadvantage.

In 2018, the SA Human Rights Commission reported on public hospitals in Gauteng province denying migrants healthcare services linked to their nationality and/or legal status [15,41]. There is a failure to ensure access to preventative and treatment interventions for all in SA, undermining any single nation’s sovereign response to both HIV and COVID-19 care [12,14,15,41]. A call to action in SA is needed for the inclusion of migrants and mobile populations to help strengthen the healthcare response nationally and form ‘regional harmonisation and operate inclusive healthcare systems’ going forward, especially for vulnerable populations like migrant WLWH [12,14,15,41].

Adaptability revealed that RMMCH echoed many of these issues with the severity of disruption and was able to rebound with COVID-19 planning. The hospital's decisions to continue to tailor specific PMTCT services are embedded in a larger healthcare referral system [33,38,40,41]. Healthcare system challenges remain which include service cutbacks for provision of counselling and support and retention in care for WLWH [16]. An increased risk of loss to follow-up (LTFU) remains because of the lack of unity between healthcare systems to trace mobile women attending different facilities with a unique ID number [16].

In light of COVID-19, studies suggest a DSD integrated approach to patient care that focuses on individualised needs and the different lived experiences of HIV, to prevent future treatment interruptions [35,42,43]. One common challenge expressed by participants is the need to access ART for a longer duration of time during the pandemic. A person-directed care approach could supply healthcare services on a virtual platform, including language-sensitive information, educational materials with visuals for communication and MMD for long-term supply of ART, to reduce the number of visits for highly mobile migrants [31]. This could help build the service users’ capacity to be in ultimate charge of choosing health services in line with their values, beliefs, and needs [33,35,42,43].

### Strengths and limitations

Self-reporting allowed participants to describe their own utilisation experiences with PMTCT in-depth and provides insights to help strengthen service provision. Self-reporting bias may be a limitation with participants reporting on adherence to ART. Some interviews conducted in Chichewa could have self-reporting bias with the assistance of a fluent speaking counsellor. People who utilised PMTCT are not representative of those who remain inaccessible, and we cannot know how their experiences may differentiate from those still absent from PMTCT care.

The vast typologies showcased by the 40 participants sampled and study results have broader applicability in mobility contexts outside Johannesburg [26]. Furthermore, the mobility typologies identified are not exhaustive of all typologies that exist. However, the transferability of using the methodology in identifying typologies for future research in migration and health is broadly applicable both in the SA population where movement between provinces and regions of the city occur. There is an opportunity for broader applicability to other countries that experience resource-constrained healthcare settings. However, limitations may be sampling in rural remote areas where limited population movement may occur when compared to urban areas.

By allowing the data to determine the typologies, rather than defining the typologies *a priori*, the study aimed to avoid the pitfalls of previous studies that assume solely that these typologies are limited to a dichotomy of citizen versus migrant [25].

Conducting qualitative field research under the COVID-19 conditions was challenging, and attempts to move interviews online proved not to be feasible to capture the nuances, body language, and observation of the participants, important in understanding participants‘ stories.

## Conclusion

The COVID-19 pandemic has shone a light on the pre-existent healthcare system challenges. Future healthcare system and pandemic preparedness emphasise the importance of adapting to the ever-changing healthcare climate in response to the needs of highly mobile WLWH.

Future research studies in migration and health should utilise the methods of not predetermining typologies *a priori* to identify and address specific challenges at both patient and healthcare system levels. RMMCH provided insight into the mobility typologies within SA utilising PMTCT programming, emphasising the importance to look at patients more holistically to better incorporate their needs and inform decision-making that occurs in government and within healthcare facilities.

To address the migrant gap in healthcare service provision, more collaboration between research, policy, and programming spaces is needed to continuously develop strategies and strengthen service provision for the inclusion of highly mobile migrant WLWH.

## Paper context

The sustainability of HIV services was a global challenge during the COVID-19 pandemic. The utilisation of PMTCT treatment and care has become more difficult for highly mobile populations due to border closures and lockdown, documentation, and xenophobic attitudes from service providers. Exploring utilisation experiences between different typologies of women allowed for tailored strategies responsive to their service needs. Differentiated service delivery models could assist in alleviating the burden on the healthcare system and patients.
